# Host sex and age influence endoparasite burdens in the gray mouse lemur

**DOI:** 10.1186/s12983-015-0118-9

**Published:** 2015-10-01

**Authors:** Anni Hämäläinen, Brigitte Raharivololona, Pascaline Ravoniarimbinina, Cornelia Kraus

**Affiliations:** Department of Sociobiology/Anthropology, Georg-August University of Göttingen, Kellnerweg 6, 37077 Göttingen, Germany; Behavioral Ecology and Sociobiology Unit, German Primate Center, Kellnerweg 4, 37077 Göttingen, Germany; Department of Paleontology and Biological Anthropology, University of Antananarivo, Antananarivo, Madagascar; Helminthiasis Unit, Institut Pasteur of Madagascar Antananarivo, Antananarivo, Madagascar; Current address: Department of Biological Sciences, University of Alberta, Edmonton, Alberta T6G 2E9 Canada

**Keywords:** Aging, Body mass, Helminth, Immunosenescence, *Microcebus murinus*, Parasite prevalence, Parasite species richness, Seasonality

## Abstract

**Introduction:**

Immunosenescence (deteriorating immune function at old age) affects humans and laboratory animals, but little is known about immunosenescence in natural populations despite its potential importance for population and disease dynamics and individual fitness. Although life histories and immune system profiles often differ between the sexes, sex-specific effects of aging on health are rarely studied in the wild. Life history theory predicts that due to their shorter lifespan and higher investment into reproduction at the expense of immune defences, males might experience accelerated immunosenescence. We tested this hypothesis by examining sex-specific age trajectories of endoparasite burden (helminth prevalence and morphotype richness measured via fecal egg counts), an indicator of overall health, in wild gray mouse lemurs (*Microcebus murinus*). To account for potential interactions between seasonality and host sex or age we examined the predictors of parasite burdens separately for the dry and rainy season.

**Results:**

Contrary to the prediction of immunosenescence, parasite prevalence and morphotype richness decreased at old age in the dry season, indicating acquired immunity by older animals. This pattern was primarily caused by within-individual decline in parasite loads rather than the earlier mortality of highly parasitized individuals. With the exception of an increasing cestode prevalence in males from yearlings to prime age in the rainy season, no evidence was found of male-biased ageing in parasite resistance. Besides this sex*age interaction, host age was uncorrelated with rainy season parasite loads. Seasonality did not affect the overall parasite loads but seasonal patterns were found in the predictors of parasite prevalence and morphotype richness.

**Conclusions:**

These results provide rare information about the age-related patterns of health in a wild vertebrate population and suggest improvement rather than senescence in the ability to resist helminth infections at old age. Overall, males appear not to suffer from earlier immunosenescence relative to females. This may partially reflect the earlier mortality of males, which can render senescence difficult to detect. While helminth infections are not strongly associated with survival in wild gray mouse lemurs, parasite load may, however, reflect overall good phenotypic quality of long-lived individuals, and is a potential correlate of fitness.

**Electronic supplementary material:**

The online version of this article (doi:10.1186/s12983-015-0118-9) contains supplementary material, which is available to authorized users.

## Introduction

Efficient immune system function can bring about health and survival benefits and thereby enhance fitness. However, due to limited resources and competing needs, immune defenses may not always be sustained at the optimal level to efficiently eradicate pathogens and parasites [1]. Resource allocation to the immune system depends on extrinsic factors including pathogen encounter rates, resource availability and energetic demands set by the environment, as well as intrinsic factors such as physical condition, life history stage, sex and age of the individual [2–4].

The functioning of the immune system changes as a function of age, from development of adaptive immunity beginning at birth to the deterioration of the system at old age [5–9]. The latter phenomenon is known as immunosenescence, and is characterized by a remodeling of the immune system, including a down-regulation of type Th2 immunity, which is involved in parasite resistance [8]. On the other hand, older animals are more likely to have developed adaptive immunity against recurring parasites [2, 10] (but see [7]), hence individuals in good enough physical condition at old age might be able to partially counter the effects of immunosenescence. Most of the evidence for immunosenescence comes from studies on humans and laboratory animals, but a few studies have also demonstrated its occurrence in natural populations [11–14]. Age-related improvement in disease resistance has also occasionally been described in the wild [10, 15, 16] but may be followed by a terminal decline in health at very old age (*e.g.* [10]). However, assessing immunosenescence in wild animals is often hampered by the reduced survival of the individuals with poor immune defenses. Aging can be masked at the population level by the “selective disappearance” of lower quality individuals, leading to more slowly declining or even apparently improving values at old age when examining cross-sectional data (*e.g.* [17]).

Among individuals, host sex is one of the most important determinants of the immune function profile [18]. In mammals, a male bias in parasite infection rates is common [19]. Ultimately, sex differences in immune responses are thought to stem from sex-specific life history optimization and the associated trade-offs between immune function and reproductive investment. Males typically benefit from optimizing reproductive effort in their prime reproductive age, whereas female fitness is generally improved by a longer reproductive lifespan due to their higher investment in each produced offspring. These constraints can lead females to invest more into health maintenance to enhance their longevity, whereas males more likely sacrifice health and lifespan for improved competitive success [20–22], potentially with the consequence of more rapid immunosenescence in males. Little is known about sex-specific patterns of immunosenescence beyond human studies, but existing research has indeed found a slower rate of decline in several immunological parameters in females relative to males in mammals (human: [23, 24], macaque: [25], rat: [26]) and at least some invertebrates (cricket: [27]), whereas immune responses seem to be stronger in males throughout life or similar for the sexes in birds (zebra finch: [28], barn swallow: [29]). Studies examining sex differences in immunosenescence in natural populations are scarce, but likewise suggest an overall female advantage in mammals [30–32].

Gastrointestinal parasite load is often used as a proxy of general health in natural populations as it can be monitored in a minimally invasive manner via fecal egg counts. While many endoparasites evoke only moderate clinical symptoms, they may nevertheless incur substantial energetic costs due to immune defense investment required to counter the infection [33, 34]. These costs are amplified by poor host condition due to a low nutritional status [34] or other stressors [35]. Perhaps due to such demands on limited resources, trade-offs have been discovered between parasite resistance and reproductive performance [1, 36, 37] as well as the rate of immunosenescence [38]. Parasite infection may also expose the individual to a higher risk of predation [39, 40]. Furthermore, macroparasite infections may alter the animal’s chance of being infected further by other pathogens and parasites [41–44] and infection by several parasite species strains the host system more than infection by one species only [45–47]. Hence, parasite infections have potentially far-reaching consequences for host survival and reproductive output.

The purpose of this study was to examine the effects of advancing age on health status as indicated by gastrointestinal endoparasite prevalence and parasite morphotype richness in a natural gray mouse lemur (*Microcebus murinus)* population. In particular, we examined whether these age effects differ for the sexes. To confirm that patterns observed in cross-sectional data were not an artifact of the selective disappearance of highly parasitized individuals, we also detailed within-individual changes in parasite loads using repeated measurements from the same individuals and assessed intermediate-term survival as a function of parasite burden.

The gray mouse lemur is a small strepsirrhine primate native to Madagascar. The average adult lifespan in the wild is 2-3 years but the maximum lifespan in the study population is 11 years [48], with females typically enjoying a longer lifespan than males. Mortality rates are high in the population, with annual turnover totaling ~40-60 % [49]. The species is used as a primate model of aging in captivity, but little evidence of senescent functional declines has been found in wild mouse lemurs [48, 50, 51]. The species is a solitary foraging, sexually monomorphic and mainly arboreal omnivore. The strong seasonality of the habitat (dry season ~ April-October, rainy season ~ November-March) leads to seasonal differences in food and water availability, diet [52], body mass [48] and behavior, which may influence overall parasite loads [53]. The effects of seasonality on parasite loads may differ for the sexes. Besides the characteristic life history differences associated with reproductive activities (males compete for females, females care for the young), female-biased nest sharing [54, 55] and allogrooming may influence sex-specific parasite loads. Most adult females use regular torpor through much of the dry season [56, 57], whereas most males remain active throughout the year. Males roam over large areas for receptive females in the mating season in October-November [58, 59]. In this season, the males’ testosterone levels reach their annual peak [60, 61], and roaming behavior may expose them to more infectious stages of parasites. Seasonality of parasite infections may also interact with host age if the old are less able to cope with the increased environmental stressors posed by the dry season [51].

Based on the predictions of life history theory and the species’ ecology, we made the following predictions:

- In reflection of immunosenescence, aging should lead to an increasing parasite prevalence and morphotype richness. Older animals might especially suffer lowered parasite resistance in the energetically demanding dry season, leading to stronger age effects in this season.
- Senescent increase in parasite burden might occur faster or earlier in males due to their shorter life expectancy and the potentially associated lower investment in self-maintenance [20, 21, 62]. Accordingly, males were expected to exhibit higher overall parasite prevalence and morphotype richness as commonly found in mammals [19, 63–65].
- Since body condition can negatively influence immune defenses, parasite prevalence and morphotype richness should correlate negatively with body mass especially in the lean, dry season.
- Parasites might be generally more prevalent in the dry season when host body mass is lowest, although some morphotypes may be more prevalent in rainy season due to seasonal fluctuation in the abundance of parasite infective stages.
- The survival of more parasitized individuals should be reduced relative to unparasitized mouse lemurs. This might lead to an apparent absence of senescence at the population level. However, if parasite resistance declines with age, repeated sampling of the same individuals should indicate this trend.

## Materials and methods

### Sample collection and analysis

Our study population (locally known as “N5”) of gray mouse lemurs has been monitored since 2002 by regular live capturing at the CNFEREF/Kirindy study site in a dry deciduous forest in western Madagascar. All individuals are sexed, aged, and individually, permanently marked with a subcutaneous transponder (Trovan EURO ID, Germany) at first capture, and weighed monthly at subsequent captures (precision ± 1 g). Since most individuals are captured in their first year of life (age estimates confirmed by morphometrics), the ages of all individuals can be estimated with a high level of accuracy. The capture and handling protocols have been detailed in *e.g.* [52, 66]. Sampling for this study was done from animals captured in September - November 2010 and 2012 (transition phase from dry season to rainy season, hereafter “dry season”) and in March – May 2011 and 2012 (transition from rainy to dry season, hereafter “rainy season”). These are the months when both sexes are active and most easily trappable, but the risk of capturing heavily pregnant or lactating females is low. We restricted these analyses to sexually mature individuals [67], excluding juveniles of the season from the rainy season sample. In total, we thus acquired 470 samples from 151 individuals aged 1-10 years (range of samples per individual = 1-13, mean = 3.1, median = 2; 95 individuals contributed more than 1 sample). A total of 253 samples were collected in the two dry seasons (females *N* = 148, males *N* = 105) and 217 samples in the two rainy seasons (females *N* = 148, males *N* = 69).

Fecal samples were collected from captured animals during handling or from cleaned traps. Fresh feces (1 – 4 pellets) were weighed (range: 0.01 – 2.28 g) and homogenized in 10 % formaldehyde in eppendorf tubes. The samples were analyzed at the Institut Pasteur de Madagascar by trained laboratory technicians using the Ritchie’s formol-ether concentration method [68]. Parasite egg and cyst morphotypes found via microscopic examination of fecal smears were identified to the closest genus, in accordance with previously described identification criteria [69–71].

From these fecal smears, we assessed the prevalence of each parasite egg morphotype (Table 1) and calculated parasite morphotype richness (at the genus level) as the total number of distinct egg types in the sample. While fecal egg counts are the most commonly used method in studying parasite infections, the method has been criticized for its potential inaccuracy, as parasite egg shedding rates vary over time and a given sample may not always contain the eggs of a parasite that is nevertheless present in the host [72]. This means that we may have missed some parasites and therefore sometimes underestimated the overall parasite loads or morphotype richness in a sample. However, assuming that the error is similar across groups, this method can serve as a means of comparing parasite loads between groups (*e.g.* hosts of different sexes or ages). To reduce the uncertainty due to the fecal egg count method, we elected to concentrate on the conservative measures of prevalence and morphotype richness. Formal analysis of the intensity of infection was not undertaken because of the difficulty of inferring intensity from fecal egg counts without the appropriate validations on temporal variation in the egg shedding rates of parasites (*e.g.* [72–74]). Such validations would typically include counts of adult worms in the gut and investigations of parasite fecundity and life histories, which were not possible in the framework of this study.

**Table 1:** Morphotypes of gastrointestinal helminth parasites found in *Microcebus murinus* in Kirindy forest

Phylum	Family	Genus	Transmission route	Prevalence
Nematoda	Ascaridida	*Ascaris* sp.	Direct	0.6 % (3)
	Subuluridae	*Subulura* sp.	Indirect ^a^	29.5 % (141)
	Capillaria	*Capillaria* sp.	Indirect ^a^	0.6 % (3)
	Oxyuridae	*Lemuricola* sp.	Direct	1.2 % (6)
		*Oxyuridae* sp.	Direct	1.5 % (7)
	Strongylida	*Oesophagostomum* sp.	Direct	1.9 % (9)
		*Strongylida* sp.	Direct	0.2 % (1)
	Trichuridae	*Trichuris* sp.	Direct	11.5 % (55)
Cestoda	Hymenolepididae	*Hymenolepis* sp.	Indirect ^a^	29.5 % (141)
Trematoda	Fasciolidae	*Fasciolidae* sp.	Indirect ^b^	0.2 % (1)
	Heterophyidae	*Metagonimus* sp.	Indirect ^b^	1.2 % (6)
	Opistorchiidae	*Opisthorchis* sp.	Indirect ^b^	0.2 % (1)

^a^insect intermediate host

^b^one or more intermediate hosts, first intermediate host typically snail

Prevalence indicated as % and number (in brackets) of infected samples. The taxonomy, transmission routes and life history characteristics are unstudied in all parasites carried by gray mouse lemurs, but can be conjectured based on data available on related parasite species [71]

### Ethical standards

All research reported in this study complied with animal care regulations and applicable national laws of Madagascar and was approved by the Ministère de l’Environment et des Eaux et Fôrets, MINEEF. The study adhered to guidelines provided by the Association for the Study of Animal Behaviour (ASAB) and the Animal Behavior Society (ABS).

### Statistics

#### General modeling details

Throughout, we assessed parasite burden via the prevalences of the three most commonly observed helminth morphotypes (*Subulura*, *Trichuris* and *Hymenolepis*, see Table 1) as well as morphotype richness (count of distinct helminth egg morphotypes present in the sample). The morphotypes were analyzed separately due to their potentially differing clinical effects and seasonal fluctuations in their relative prevalence that might mask patterns of infection. Statistical analyses of the less common morphotypes (recovered in <10 infected samples) were not possible due to the extremely low number of infections, hence these data were used only in assessing parasite morphotype richness. Analyses of “overall parasite prevalence”, *i.e.* infection by any of the discovered parasite morphotypes are shown in the Additional file 1: Tables S4 to S7, Figures S2 and S3.

Parasite prevalence was modeled using generalized linear mixed models (GLMM) with a binomial error structure and a logit link function, and morphotype richness was modeled using a GLMM with a Poisson error distribution and a log-link function. All GLMMs were built using the R package *lme4* [75]. Age, body mass and sample mass were each log-transformed prior to analyses to achieve normality and linearity. Age and body mass were further scaled and centered prior to analyses to improve the interpretation of interaction terms and relative effect sizes [76]. Log-transformed sample mass was added as a covariate to all models to account for the influence of fecal mass on the likelihood of detecting parasite eggs. Individual identity was included as a random factor in all GLMMs to account for repeated sampling of the same individuals. For each model, a random intercept model was selected based on lower AIC-values over a random slope and intercept structure.

Satterthwaite estimation was used to compute *P*-values (lmerTest-package [77]). Marginal and conditional *R*^*2*^-scores [78] were computed to assess model fit. R^2^_marginal_ describes the variance attributable to the fixed effects alone, whereas R^2^_conditional_ reflects the combined proportion of variance caused by both fixed and random effects. Variation accountable to the random factor (individual identity) is reported throughout as σ^2^. All analyses were performed in program R version 3.1.1 [79].

#### Predictors of parasite prevalence and morphotype richness in the complete data set

We first tested for seasonal differences in the prevalence of each of the common morphotypes as well as morphotype richness using GLMMs. Each of these models included the fixed factors season, sex, sex by season interaction, log-transformed sample mass and year of sampling, and individual identity as a random factor. The results of these models are shown in the Additional file 1: Table S1. Because of the complexity of the ensuing models, models were built separately for the dry and the rainy season to explore the effects of age, sex and body mass on the prevalences of each the three most common helminth morphotypes and morphotype richness.

The initial models of parasite prevalence and morphotype richness each included the fixed terms sex, age (range: 1-10 years) and their interaction, body mass (range: 36 - 131 g), year of sampling (2010 – 2012) and sample mass (range: 0.012 – 2.282 g). Throughout, when the interaction of age*sex and/or body mass exceeded a significance threshold of *P >* 0.1, the terms were removed from the model to avoid overparameterization [80] and to improve sample size (not all samples could be matched to body mass measurements within a few days of the sample in the interest of minimizing occasions of animal handling). The sample sizes for each model are indicated in the result tables. The final, reduced models are reported below and the full models including all terms are included in the Additional file 1: Table S2. The morphotype richness data were not overdispersed (goodness of fit tested in R package *aods3* [81]) or zero-inflated (tested with likelihood ratio tests using R package *glmmADMB* [82, 83]).

#### Within-individual change and selective mortality

Finally, we assessed the relative contributions of selective mortality of parasitized individuals and age-related change in parasite load on the observed patterns. For this, we re-ran the models for parasite prevalence and morphotype richness using only longitudinal data (repeated measures from the same individuals sampled over multiple seasons). These data were split by sex rather than by season in order to maximize the number of samples per individual in each model. The models were run separately for the sexes (*N =* males: 55 samples/20 individuals, females: 99 samples/40 individuals) and including season of sampling as well as initially the interaction terms season*age and season*body mass(for full model see Additional file 1: Table S3). These interaction terms were dropped from the model where they had non-significant effects.

To estimate whether parasite load predicts intermediate term survival, we assessed the 6 month survival probability of individuals as a function of their overall infection rate and morphotype richness. Apparent 6 month survival was extracted from the long term capture data for all individuals sampled at least once in either dry season (2010/2012) or in the 2012 rainy season (no data were available for the season following the 2011 rainy season). The individual was assigned survival status 1 if it was recaptured in the subsequent 3 month field season (*i.e.* 2011/2013 rainy season respectively for dry seasons 2010/2012 or 2012 dry season for 2012 rainy season). Individuals that were not recaptured within the next season were assumed to have died. While capture probabilities could not be accounted for in this study to conduct rigorous capture-mark-recapture survival analyses, capture probabilities in another nearby subpopulation has previously been estimated at > 0.5 [49]. The data were restricted to one seasonal sample (first sample of the season) from each individual (*N =* 37 individuals in 2010 dry season, 73 individuals in 2012 dry season, 29 individuals in 2012 rainy season, in total 92 females and 47 males). Generalized linear models (binomial GLM) were built to model the influences of overall parasite prevalence and morphotype richness on survival. These models of apparent survival contained the fixed effects sex, age, season (dry season 2010 or 2012 or rainy season 2012; known differential survival probability over the dry and rainy season [49]) and an indicator of parasite burden (overall infection or morphotype richness). For 13 individuals that were sampled in both 2010 and 2012 dry seasons, only data for one of the seasons (randomly assigned) was used in this model.

## Results

### Seasonal characteristics of the parasite community

In total, 12 different helminth egg morphotypes were identified (Table 1), with morphotype richness ranging from 0 to 4 per sample. At least one parasite morphotype was found in 55.7 % (262) of the 470 samples. The overall infection rate, *i.e.* proportion of samples infected by one or more parasite morphotypes, was essentially identical in the dry (55.7 %) and in the rainy (55.8 %) season. *Trichuris* and *Hymenolepis* were significantly more prevalent in the dry season (*Trichuris* dry: 16.2 %, rainy: 6.5 % of samples infected; *Hymenolepis* dry: 32.4 %, rainy: 26.3 %), whereas *Subulura* was significantly more prevalent in the rainy season (dry: 26.1 %, rainy: 33.6 %). The males generally carried more parasites than females (significantly so for *Subulura* and morphotype richness) in the dry season but this sex difference was negligible in the rainy season (Additional file 1: Table S1).

### Predictors of morphotype prevalence

**Fig. 1:**
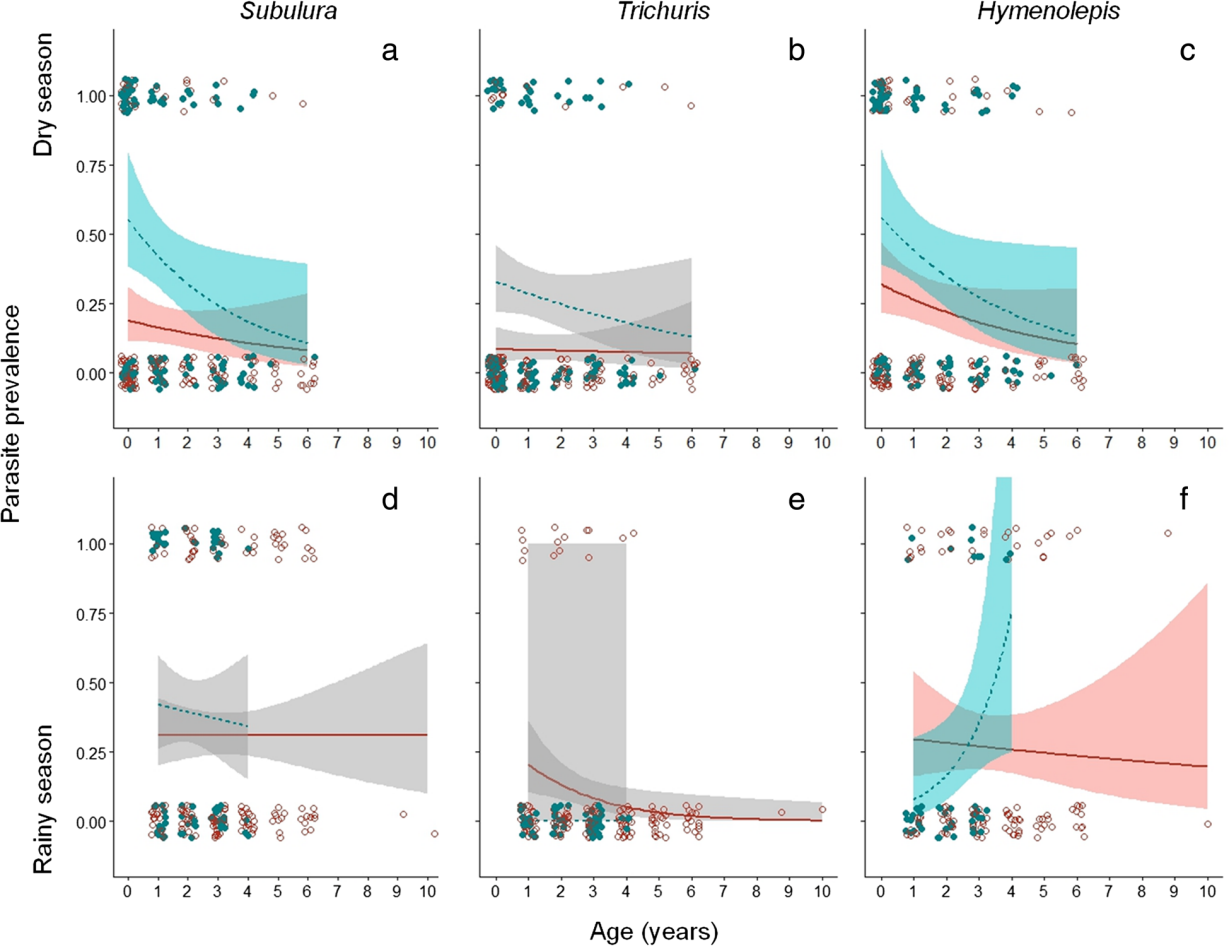
Seasonal prevalence of the three common parasite egg morphotypes. Prevalences (probability of infection) shown for *Subulura* (**a**) dry season and (**d**) rainy season; *Trichuris* (**b**) dry and (**e**) rainy season; and *Hymenolepis* (**c**) dry and (**f**) rainy season in samples from gray mouse lemurs in Kirindy forest. Shown are all data points (with jitter introduced to the discrete variables for ease of interpretation) for males (solid symbol) and females (open symbol), and loess-smoothed prediction lines and 95 % confidence bands for age effects for males (dashed line) and females (solid line). Significant age effects (a and c, trajectories not significantly different for the sexes), and a significant age*sex interaction (**f**) shown with colored confidence bands, non-significant relationships with gray confidence bands. See text and Table 2 for details

Contrary to our prediction of immunosenescence, host age was negatively associated with the prevalence of eggs of the *Subulura* and *Hymenolepis* families, but not *Trichuris*, in the dry season (Table 2, Fig. 1). More of these parasites were found in males and in young individuals relative to females and older individuals. In line with our prediction of earlier immunosenescence in males, a significant sex*age interaction was found for *Hymenolepis* in the rainy season with older males exhibiting higher infection rates, but no other models indicated a significant effect of the sex*age interaction term (Table 2, Fig. 1). *Trichuris*-type eggs were found in the rainy season only in samples collected in year 2012 from female hosts, with all other samples being negative. No age or sex effects were found in the rainy season for *Subulura.*

**Table 2:** Predictors of parasite prevalences and morphotype richness in the dry and rainy season

		Dry season				Rainy season			
		β	SE	z	P	β	SE	z	P
*Subulura*	Intercept	−0.983	0.610	−1.610	0.107^a^	−2.047	0.668	−3.063	**0.002** ^**b**^
prevalence	Sex (ref. female)	1.481	0.322	4.600	**<0.001**	0.388	0.423	0.919	0.358
	Age	−0.500	0.614	−3.049	**0.002**	−0.232	0.299	−0.775	0.438
	Year	−0.835	0.400	−2.087	**0.037**	1.841	0.441	4.179	**<0.001**
	Sample mass	0.218	0.203	1.078	0.281	0.057	0.287	0.199	0.842
	Body mass	-	-	-	-	-	-	-	-
	Sex*Age	-	-	-	-	-	-	-	-
*Trichuris*	Intercept	−7.399	2.861	−2.586	**0.010** ^**c**^	−20.976	12.078	−1.737	0.082 ^d^
prevalence	Sex (ref. female)	1.870	1.612	1.160	0.246	-	-	-	-
	Age	−0.248	0.735	−0.338	0.735	4.557	5.139	0.887	0.375
	Year	0.021	1.391	−0.015	0.988	-	-	-	-
	Sample mass	0.245	0.637	0.385	0.700	−1.803	1.549	−1.164	0.245
	Body mass	2.103	1.239	1.696	0.090	-	-	-	-
	Sex*Age	-	-	-	-	-	-	-	-
*Hymenolepis*	Intercept	−1.618	0.676	−2.393	**0.017** ^**e**^	0.512	0.797	0.642	0.521^f^
prevalence	Sex (ref. female)	0.736	0.331	2.224	**0.026**	−1.395	0.651	−2.145	**0.032**
	Age	−0.844	0.197	−4.284	**<0.001**	0.029	0.391	0.074	0.941
	Year	0.595	0.441	1.348	0.178	−0.638	0.471	−1.355	0.175
	Sample mass	−0.067	0.198	−0.341	0.733	0.253	0.341	0.744	0.457
	Body mass	3.883	1.220	3.184	**0.001**	−2.802	1.228	−2.282	**0.023**
	Sex*Age	-	-	-	-	2.484	0.991	2.507	**0.012**
Morphotype	Intercept	−0.210	0.313	−0.669	0.503^g^	0.013	0.393	0.033	0.973^h^
richness	Sex (ref. female)	0.722	0.165	4.383	**<0.001**	−0.408	0.269	−1.516	0.130
	Age	−0.341	0.094	−3.632	**<0.001**	−0.157	0.200	−0.787	0.432
	Year	−0.222	0.200	−1.106	0.269	0.428	0.242	1.769	0.077
	Sample mass	0.133	0.098	1.353	0.176	0.132	0.174	0.756	0.450
	Body mass	0.272	0.143	1.901	0.057	−0.431	0.173	−2.496	**0.013**
	Sex*Age	-	-	-	-	0.705	0.419	1.682	0.092

^a^ *N* = 253 / 113 /66 (samples/ individuals/ infected); R^2^_marginal_ = 0.224, R^2^_conditional_ = 0.224, σ^2^ < 0.001

^b^ *N* = 217/ 93/121; R^2^_marginal_ = 0.176, R^2^_conditional_ = 0.281, σ^2^ = 0.481

^c^ *N* = 253/ 113/41; R^2^_marginal_ = 0.025, R^2^_conditional_ = 0. 968, σ^2^ = 96.900

^d^ *N* = 148/ 66/14; R^2^_marginal_ = 0.014, R^2^_conditional_ = 0. 995, σ^2^ = 649.600; infections observed only in females in year 2012, hence non-convergence if year and sex appear as fixed terms

^e^ *N* = 235/ 110/82; R^2^_marginal_ = 0.206, R^2^_conditional_ = 0.253, σ^2^ = 0.206

^f^ *N* = 154/ 88/57; R^2^_marginal_ = 0.147, R^2^_conditional_ = 0.147, σ^2^ < 0.001

^g^ *N* = 235 samples /110 individuals, R^2^_marginal_ = 0.212, R^2^_conditional_ = 0.274, σ^2^ = 0.006

^h^ *N* = 154 samples/88 individuals, R^2^_marginal_ = 0.192, R^2^_conditional_ = 0.192, σ^2^ < 0.001

Predictions are based on the final, reduced models of the prevalences of the three common parasite morphotypes and morphotype richness after dropping the terms age*sex and body mass where they had non-significant (*P* > 0.1) effects (empty cells). P-values for significant predictors at significance threshold *P* < 0.05 shown in bold

The prevalence of cestode (*Hymenolepis*) eggs was, unexpectedly, associated with higher body mass in the dry season, and no other morphotype-specific models indicated a significant influence of body mass. In addition to the season-specific characteristics of morphotype prevalence, a significant effect of the year of sampling was found in most morphotype-specific models (Table 2), indicating substantial temporal fluctuations in the gray mouse lemurs’ parasite communities.

### Predictors of parasite morphotype richness

**Fig. 2:**
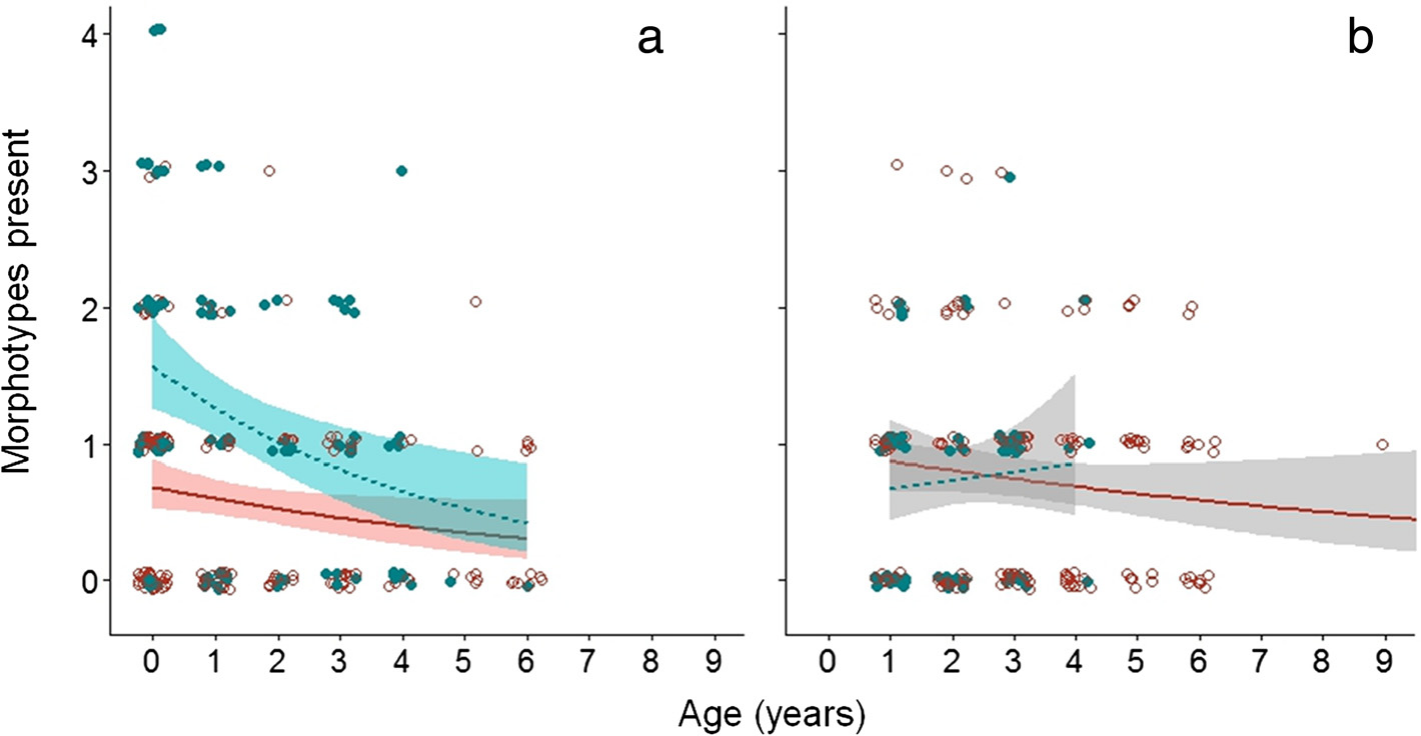
Parasite morphotype richness as a function of age in gray mouse lemurs. Predictions for morphotype richness in (**a**) dry and (**b**) rainy season shown for males (solid symbols, dashed line) and females (open symbols, solid line). Lines indicate loess smoothers of the age effects (significant decline (in color) in dry season, non-significant (gray-scale) in rainy season) based on the final model in each season (Table 2), and shaded areas indicate 95 % confidence intervals. Morphotype richness was significantly higher in males relative to females in the dry season, whereas no significant sex effect was found in the rainy season. The sex*age interaction was non-significant at the *P* > 0.05 threshold in both seasons. Both age and morphotype richness are based on discrete measures but jitter was introduced to improve interpretability

Parasite morphotype richness ranged from 0 to 4 morphotypes in infected samples. Of the 262 infected samples, 32.8 % (86) contained eggs of more than one morphotype. Based on the final model (Table 2, Fig. 2), morphotype richness declined significantly with age in the dry season but not in the rainy season. Males carried on average twice as many parasite morphotypes as females in the dry season (average for males: 1.2, females: 0.6 morphotypes), whereas no significant sex effect was found in the rainy season (average for both: 0.7 morphotypes). Body mass had a negative effect on morphotype richness in the rainy season, but had the opposite effect in the dry season, likely due to the association of body mass with *Hymenolepis* infections (Table 2).

### Within-individual change and apparent survival

Analyses of the longitudinal data indicate no statistically significant effects of age on parasite burden (Table 3). However, similarly to the results outlined above for the full data set, the estimates from the models point toward a declining trend in parasite prevalence as well as parasite morphotype richness with age, but also with increasing body mass (Table 3, Additional file 1: Figure S1 ). The slopes of the age effect varied in magnitude between the longitudinal and cross-sectional data sets, the response generally being of a lower magnitude in the longitudinal models relative to the full data set (compare β-values in Tables 2 and 3). However, direct comparison is not possible due to the different structures of the data sets (splitting by season or sex) and the subsequently different predictors in the models.

**Table 3:** Predictors of parasite burden in males and females based on the longitudinal data

		Males				Females			
		β	SE	z	P	β	SE	z	P
*Subulura*	Intercept	−0.927	0.477	−1.945	0.052 ^a^	−2.927	0.659	−4.440	**<0.001** ^b^
Prevalence	Season (ref. dry)	0.249	0.597	0.417	0.679	2.137	0.895	2.388	**0.017**
	Age	−0.205	0.354	−0.580	0.562	0.004	0.306	0.014	0.989
	Body mass	-	-	-	-	−1.013	0.460	−2.204	**0.028**
	Season *Age	-	-	-	-	-	-	-	**-**
	Season *Body mass	-	-	-	-	-	-	-	**-**
*Trichuris*	Intercept	-	-	-	-^d^	−2.219	0.529	−4.192	**<0.001** ^c^
prevalence	Season (ref. dry)	-	-	-	-	−0.931	0.908	−1.026	0.305
	Age	-	-	-	-	−0.455	0.351	−1.297	0.195
	Body mass	-	-	-	-	-	-	-	-
	Season *Age	-	-	-	-	-	-	-	-
	Season *Body mass	**-**	-	-	-	-	-	-	-
*Hymenolepis*	Intercept	−2.439	1.299	−1.878	0.060 ^e^	−2.119	0.631	−3.359	**<0.001** ^f^
prevalence	Season (ref. dry)	−0.226	0.873	−0.259	0.796	0.785	0.604	1.300	0.194
	Age	−0.851	0.784	−1.085	0.278	−0.204	0.332	−0.613	0.540
	Body mass	-	-	-	-	-	-	-	-
	Season *Age	-	-	-	-	-	-	-	-
	Season *Body mass	-	-	-	-	-	-	-	-
Morphotype	Intercept	−0.430	0.267	−1.613	0.107 ^g^	−1.259	0.312	−4.033	**<0.001** ^h^
richness	Season (ref. dry)	0.020	0.332	0.061	0.952	0.926	0.449	2.063	**0.039**
	Age	−0.188	0.192	−0.982	0.326	−0.102	0.155	−0.658	0.511
	Body mass	-	-	-	-	−0.419	0.221	−1.897	0.058
	Season *Age	-	-	-	-	-	-	-	-
	Season *Body mass	-	-	-	-	-	-	-	-

^a^ *N* = 55 samples/20 individuals, R^2^_marginal_ = 0.065, R^2^_conditional_ = 0.065, σ^2^ < 0.001

^b^ *N* = 95 samples/40 individuals, R^2^_marginal_ = 0.149, R^2^_conditional_ = 0.149, σ^2^ < 0.001

^c^ *N* = 99 samples/40 individuals, R^2^_marginal_ = 0.143, R^2^_conditional_ = 0.143, σ^2^ < 0.001

^d^No *Trichuris* infections in males

^e^ *N* = 55 samples/20 individuals, R^2^_marginal_ = 0.098, R^2^_conditional_ = 0.428, σ^2^ = 1.897

^f^ *N* = 99 samples/40 individuals, R^2^_marginal_ = 0.033, R^2^_conditional_ = 0.304, σ^2^ = 1.282

^g^ *N* = 49 samples/20 individuals, R^2^_marginal_ = 0.084, R^2^_conditional_ = 0.108, σ^2^ = 0.023

^h^ *N* = 95 samples/40 individuals, R^2^_marginal_ = 0.095, R^2^_conditional_ = 0.132, σ^2^ = 0.051

Predictions are based on the final models using repeated measures from the same individuals, after dropping interaction terms and body mass when non-significant at threshold *P* > 0.1 (empty cells). P-values for statistically significant results at the significance threshold *P* < 0.05 in bold

**Fig. 3:**
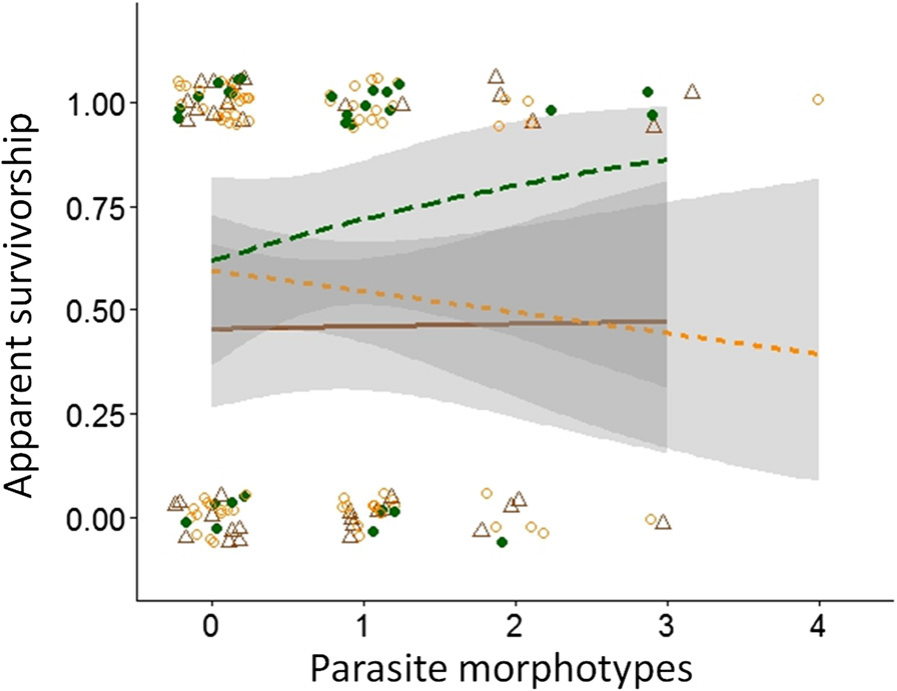
Apparent survival of gray mouse lemurs is not significantly influenced by morphotype richness (Table 4). Apparent survival to next season (~6 months, based on capture data) as a function of parasite infections in the 2010 dry season (open brown triangle & solid line), 2012 dry season (open orange circles & dotted line) and the 2012 rainy season (filled green circles & dashed line). Shown are all data points (with jitter introduced to the discrete variables for ease of interpretation) and season-specific loess-smoothed prediction lines and 95 % confidence bands based on a binomial GLM

The average apparent survival probability to next season (*i.e.* ~6 months, based on capture data) of individuals infected by one or more parasite morphotypes was 0.47 ± 0.10 in the dry season and 0.66 ± 0.11 in the rainy season, whereas for uninfected individuals the same survival probabilities were 0.55 ± 0.10 and 0.72 ± 0.10, respectively. This tentatively suggests that individuals infected with gastrointestinal parasites may experience a slightly lower survival probability, but this difference is not statistically significant (Table 4). Within infected animals, morphotype richness had no influence on apparent survival, as the survival probability of individuals excreting one morphotype was 0.57 ± 0.09 relative to 0.57 ± 0.16 for those individuals carrying 3-4 different morphotypes (Fig. 3, Table 4).

**Table 4:** Parasite load as a predictor of survival to next season

		β	SE	z	P
*Subulura* prevalence^a^	Intercept	−0.121	0.359	−0.338	0.736
	*Subulura* prevalence	−0.080	0.422	−0.189	0.850
	Sex (ref. female)	−0.043	0.387	−0.111	0.912
	Age	0.035	0.182	0.194	0.846
	season 2012 dry	0.412	0.413	0.997	0.319
	season 2012 rainy	0.939	0.533	1.762	0.078
*Trichuris* prevalence^b^	Intercept	−0.124	0.349	−0.354	0.724
	*Trichuris* prevalence	−0.155	0.506	−0.306	0.760
	Sex (ref. female)	−0.052	0.371	−0.140	0.889
	Age	0.039	0.179	0.218	0.827
	season 2012 dry	0.422	0.409	1.033	0.302
	season 2012 rainy	0.939	0.533	1.763	0.078
*Hymenolepis* prevalence^c^	Intercept	−0.261	0.356	−0.734	0.463
	*Hymenolepis* prevalence	0.637	0.427	1.494	0.135
	Sex (ref. female)	−0.050	0.371	−0.134	0.894
	Age	0.085	0.183	0.467	0.640
	season 2012 dry	0.390	0.412	0.945	0.344
	season 2012 rainy	0.870	0.538	0.618	0.106
Morphotype richness^d^	Intercept	−0.140	0.374	−0.374	0.708
	Morphotype richness	−0.001	0.206	−0.003	0.997
	Sex (ref. female)	−0.065	0.374	−0.175	0.861
	Age	0.042	0.184	0.226	0.821
	season 2012 dry	0.423	0.411	1.031	0.303
	season 2012 rainy	0.937	0.533	1.758	0.079

*N* = 139 individuals

^a^Nagelkerke’s R^2^ = 0.035

^b^Nagelkerke’s R^2^ = 0.036

^c^Nagelkerke’s R^2^ = 0.056

^d^Nagelkerke’s R^2^ = 0.035

Season reference level is dry season 2010

## Discussion

In this study, we tested the hypothesis that aged wild animals and particularly males would suffer from a high parasite burden due to sex-specific immunosenescence. To this end, we investigated the seasonal effects of age and sex on the endoparasite burden of gray mouse lemurs. Contrary to our prediction of higher parasite prevalence and morphotype richness at old age, age effects were either absent or negative for nematodes, indicating a lower parasite burden at old age. A positive association between age and cestode prevalence was only found for males in the rainy season, offering limited support for the prediction of sex-specific ageing. Overall, we found evidence of higher parasite prevalence and morphotype richness in males as expected based on the likely trade-offs between reproductive investment and immunosuppression, or male-biased parasite encounter rates.

### Parasite community of gray mouse lemurs in Kirindy forest

Out of the 12 distinct helminth egg morphotypes found in this study, three morphotypes (the nematodes *Subulura* and *Trichuris* and a cestode *Hymenolepis*) accounted for the vast majority of the infections. Clinical effects of the specific parasites infecting mouse lemurs are unknown but their congeners generally induce at most mild pathogenic effects [53, 71], although particularly heavy infection or infection by multiple parasite species may be detrimental. Morphotype richness may be indicative of increasing pathogenicity and weakened individuals may suffer from the additional energetic demands of fighting an infection, leading to reduced reproductive output or health failure.

### Predictors of parasite prevalence and morphotype richness

#### Older animals have lower parasite burdens

Declines in immune system function are commonly found at old age, but acquired immunity may counteract some of the detrimental effects [84]. In this study, parasite burdens (morphotype richness as well as the prevalence of one nematode and one cestode) were lower in older animals in the dry season, suggesting acquired immunity rather than a senescent decline in parasite resistance. This seasonal pattern is contrary to our prediction that aged animals might suffer particularly high parasitism in the dry season due to impaired coping with the energetically demanding conditions (see also [51]). In the rainy season, a positive relationship between age and cestode prevalence was found for males but not females, and no age effects were evident in morphotype richness or the prevalence of other parasite morphotypes. Therefore, in accordance with previously described patterns of functional aging in the same population [48], it appears that the oldest animals are generally not in poorer condition than younger animals, and rather benefit from acquired immunity that leads to lower parasite burdens [2].

An absence of senescent declines in functioning could also follow from selective mortality of individuals with weaker immune defenses at an earlier age. Our data suggest that parasitized individuals may suffer a slightly reduced (albeit statistically non-significant) chance of surviving to the next season. Meanwhile, the (non-significant) effects of age continued to be negative when the data were restricted to longitudinal data only, indicating that the effects of age found in the complete data set were not solely an artifact of selective mortality. Together, these results point towards within-individual declines in parasite burden with advancing age and suggest age-related improvement rather than senescence in helminth resistance. It is also noteworthy that, although the intensity of infection was not statistically analyzed due to the difficulty of interpretation (*e.g.* [72–74]), a clear decline with age was found in the total intensity of egg shedding (total egg counts as a function of age illustrated in the Additional file 1: Figure S4).

These results are in contrast with those from red-fronted lemurs in which age had little effect on the parasite burden [85], and with reports of higher parasite burdens in older animals in *e.g.* wild and captive rodents [7, 86], yellow baboons [31], Soay sheep: [32] and brown mouse lemurs [87]. Similar to this study, indications of acquired immunity indicated by reduced parasite load at old age have been found in chacma baboons (declining species richness [15]), gibbons [16], dogs and cats [88] as well as frogs [10]. However, the prevalence of certain parasites sometimes still increases in exceptionally old individuals [10, 88], possibly reflecting terminal illness or age-associated loss of body condition.

In summary, so far, no clear patterns of immunosenescence in wild populations have emerged. The variable results might reflect differing pathogenic effects of various parasites and the immune responses they elicit, or behavioral or nutritional changes of hosts at old age that may influence encounter rates or resistance. Furthermore, it is possible that highly parasitized individuals die earlier than their more parasite resistant counterparts [89], although the results of this study suggest this effect is negligible in mouse lemurs. Further studies of the simultaneous effects of aging on health and health on survival are needed in a range of species (and a range of parasites) to clarify the significance of these parallel processes.

#### Little evidence of earlier immunosenescence in males

Our prediction of earlier immunosenescence in males was only supported by the finding of an increasing *Hymenolepis* prevalence with age in males in the rainy season. An earlier onset of decline in parasite resistance was similarly found in male Soay sheep relative to females [32]. The elevated cestode prevalence of older males in the rainy season might result from acquisition of long-lived or slowly maturing parasites [90] during the mating season, when immune defenses may be lowered due to elevated testosterone levels. However, due to the short male lifespan and high mortality during the mating season, the oldest males we sampled in the rainy season were only 4 year-olds, which is the age at which senescent declines in other measures of functioning typically start [48, 67, 91]. Therefore, the increase in parasite prevalence with age should be interpreted with caution as it may not indicate immunosenescence as much as, for instance, higher reproductive investment by older males in the preceding dry season. This might be expected based on their declining reproductive value [92] and reflect the increasing risk taking by males at advancing age [93]. One alternative explanation would be differential exposure due to dietary preferences of older males favoring potential cestode intermediate hosts, but any prey preferences remain to be demonstrated. Determining the pathogenicity of this parasite for mouse lemurs would be needed to evaluate its potential contribution to the higher male mortality in the species. The (sex-specific) fitness consequences of parasite infection rates in primates remain to be studied.

#### Males have higher parasite prevalence and carry more parasite genera

The prevalence of infection with the most common nematode and cestode morphotype as well as morphotype richness were consistently higher in males relative to females in the dry season, whereas no sex difference was found in the rainy season. These dry season results concur with the general male bias in parasite prevalence found across host taxa, the pattern being strongest for helminthiasis in mammals [19, 94], see also [95].

Explanations offered for the typical male-bias in parasitism include endocrinological and behavioral sex differences [2, 65] attributable to evolutionary mechanisms that aim to maximize the reproductive output of each sex [65]. Proximately, sex differences in parasite burden are usually ascribed to pleiotropic effects of steroid hormones, especially the potential immunosuppression associated with increased testosterone levels [27, 63–65, 94, 96, 97]. The sex bias in infection rates might also follow from differing behavioral repertoires, which may in turn be mediated by hormonal states [6]. In the late dry season when our sampling took place, the males’ testosterone levels are at their annual high [60, 61] and males roam over larger areas in search of females [98, 99], which may increase their parasite encounter probabilities [15, 100, 101]. Particularly in species where potential for intense male-male competition accompanies a polygynous mating system, as in gray mouse lemurs, males might be expected to suffer higher degrees of parasitism as a result of their higher investment in competition over self maintenance. However, contrary to our predictions, this did not consistently translate into accelerated senescence in males in our study population. This suggests that other processes are at play, potentially including early mortality of lower quality males that occurred so rapidly that it was not captured with these data.

The absence of sex difference in our rainy season data and the *Trichuris* infections exclusive to females in rainy season may reflect the effects of breeding on female parasite loads as well as the lower testosterone levels of males. Breeding has substantial energetic costs for females [102, 103], and together with the immunosuppressive effects of hormones produced during parturition and lactation [104] might lead to elevated infection rates of females in the rainy season. Furthermore, parasites that spread by direct transmission may infect females more than males due to female-biased nest sharing in the species [54, 55].

#### Body mass is associated with cestode prevalence

Since body mass broadly reflects the energetic status of an individual and, potentially, their nutritional status, it tends to be associated with the functioning of the immune response [105] and the ability to resist infection and compensate for damage caused by parasites [2]. Furthermore, parasite infections may lead to a declining body mass [4]. Consistently, body mass correlates negatively with parasitism in *e.g.* red deer [106] and pythons [14]. In this study, body mass was negatively associated with cestode prevalence in the rainy season, but surprisingly, this association was positive in the dry season (similar trends observed for morphotype richness and, to a lesser extent, *Trichuris* prevalence). The contrasting effects of body mass on cestode infection rate might reflect complex interactions of sex, age and sex-specific seasonal fluctuations [48] that could not be tested in detail due to limitations of sample size and sampling intervals.

As predicted by life history theory, males may invest any excess resources in reproduction rather than immune defences. In the dry season, the body condition effect may have significance for male fitness. Males that are in a good body condition may be able to expend more energy into mating activities, while also allocating sufficient resources to cope with a parasite infection. Even sub-clinical parasite infections have been previously linked to reduced body mass gain, changes in body composition, and reduced nutrient utilization efficiency [3], hence parasite infections may influence the males’ general health status, increase energetic expenditure and consequently increase their predation risk or affect recovery after the mating season. This might make parasitism a potential indicator of male phenotypic quality, *sensu* the Immunocompetence Handicap Hypothesis [63]. Body mass also tended to be negatively associated with parasite prevalence in females but not males in the longitudinal data (see also Additional file 1: Table S6), although this result was statistically non-significant at the *P* < 0.05 level. This trend might indicate an increased investment in self maintenance, including parasite resistance with increasing resources or improved condition in females.

## Conclusions

The parasite burden observed based on fecal samples reflects the combined outcome of several factors: the parasites the host has encountered; when and how successfully the parasite matured and reproduced within the host; and how effective the host’s immune defenses were in clearing the infection. While it is very difficult to pull apart these factors in a minimally invasive study of a wild population, we could still draw broad inferences about population level predictors of the parasite burden. We found males to have generally higher parasite infection rates than females, and the oldest animals to suffer from the lowest parasite burden within this population, suggesting a role for acquired immunity. Our results suggest that host susceptibility may govern parasite distribution in the ecologically demanding dry season, whereas in the rainy season stochastic processes have more influence on parasite distribution, as host-specific traits were less useful in predicting rainy season parasite burden. The seasonal differences in the predictors of parasite burdens are noteworthy for future studies of immunosenescence, as for example senescence may be overlooked if seasonal patterns are ignored.

While these results comprise one of the most thorough investigations into parasite loads of a lemur population in terms of the number of individuals sampled, it is only the first step towards understanding the parasite communities and variation among hosts in the parasite loads suffered. The taxonomy, life cycles and pathogenic effects of lemur parasites are virtually unstudied, as are the various host-specific determinants of parasite susceptibility and resistance. Furthermore, the study of other pathogens besides macroparasites is needed to evaluate the health of the population more comprehensively. Further longitudinal studies that monitor the age trajectories of parasite burden and survival of individuals would also be needed to confirm whether the individuals that are able to resist parasite infection enjoy any survival advantages or a higher reproductive output to evaluate the overall fitness consequences of parasites on their hosts.

## Additional file

Additional file 1:
**Additional results for Hämäläinen et al. "Host sex and age influence endoparasite burdens in the gray mouse lemur".** (PDF 875 kb)
