# Supplementary material for: Host sex and age influence endoparasite burdens in the gray mouse lemur
Source: Front Zool. 2015 Oct 1;12:25. doi: 10.1186/s12983-015-0118-9 (PMC4591582; doi:10.1186/s12983-015-0118-9)
Supplement: Additional file 1: — Additional results for Hämäläinen et al. "Host sex and age influence endoparasite burdens in the gray mouse lemur". (PDF 875 kb) [file 12983_2015_118_MOESM1_ESM.pdf]

Appendix to Hämäläinen et al.: “**Host sex and age influence endoparasite burdens in the gray mouse lemur**”

DOI: 10.1186/s12983-015-0118-9

*Sex*season interaction in parasite burden*

Table S1. The influence of seasonal and yearly fluctuations on gastrointestinal parasite infection rates and parasite morphotype richness of gray mouse lemurs in Kirindy forest based on fecal egg counts. Year of sampling reference level is 2010.

		β	SE	z	P
Subulura prevalence [a]	Intercept	-0.824	0.536	-1.537	0.124
	Sex (ref. Female)	1.460	0.340	4.298	<0.001
	Season (ref. Dry)	1.525	0.348	4.382	<0.001
	Year 2011	-2.536	0.574	-4.420	<0.001
	Year 2012	-0.808	0.396	-2.039	0.041
	Sample mass	0.183	0.163	1.122	0.262
	Sex * Season	-0.984	0.469	-2.097	0.036
Trichuris prevalence [b]	Intercept	-5.259	1.262	-4.169	<0.001
	Sex (re. Female)	2.580	1.383	1.865	0.062
	Season (ref. Dry)	-0.333	0.731	-0.456	0.649
	Sample mass	0.356	0.381	0.936	0.349
	Sex * Season	-93.930	<0.001	0.000	1.000
Hymenolepis prevalence [c]	Intercept	-1.326	0.566	-2.342	0.019
	Sex (re. Female)	0.839	0.343	2.446	0.014
	Season (ref. Dry)	-0.191	0.340	-0.565	0.572
	Year 2011	0.711	0.552	1.289	0.197
	Year 2012	0.626	0.425	1.475	0.140
	Sample mass	0.181	0.158	1.143	0.253
	Sex * Season	-0.826	0.487	-1.698	0.090
Overall prevalence [d]	Intercept	0.7455	0.498	1.496	0.135
	Sex (ref. Female)	1.215	0.330	3.684	<0.001
	Season (ref. Dry)	0.546	0.305	1.790	0.073
	Year 2011	-1.194	0.489	-2.443	0.015
	Year 2012	-0.164	0.364	-0.451	0.652
	Sample mass	0.400	0.148	2.695	0.007
	Sex * Season	-0.829	0.452	-1.832	0.067

Morphotype richness [e]	Intercept	-0.246	0.263	-0.934	0.350
	Sex (ref. Female)	0.755	0.162	4.675	<0.001
	Season (ref. Dry)	0.397	0.169	2.350	0.019
	Year 2011	-0.761	0.270	-2.817	0.005
	Year 2012	-0.180	0.189	-0.954	0.340
	Sample mass	0.116	0.077	1.496	0.135
	Sex * Season	-0.720	0.233	-3.096	0.002

N= 470 samples/151 individuals

[a] $R^2_{marginal}$= 0.164, $R^2_{conditional}$= 0.221, σ^2=0.243

[b] $R^2_{marginal}$=0.979, $R^2_{conditional}$= 0.997, σ^2=22.880

[c] $R^2_{marginal}$=0.049, $R^2_{conditional}$=0.190, σ^2=0.573

[d] $R^2_{marginal}$=0.099, $R^2_{conditional}$=0.209, σ^2=0.458

[e] $R^2_{marginal}$=0.106, $R^2_{conditional}$=0.212, σ^2=0.119

Full models of morphotype prevalence and morphotype richness prior to term reduction

Table S2. Predictors of the prevalence of the three common parasite morphotypes and morphotype richness in fecal samples collected in dry and rainy season. Predictions are based on the initial models prior to dropping non-significant terms.

		**Dry season**				**Rainy season**			
		**β**	**SE**	**z**	**P**	**β**	**SE**	**z**	**P**
Subulura	Intercept	-0.605	0.692	-0.875	0.382 [a]	-1.466	0.798	-1.836	0.066 [b]
prevalence	Sex (ref. female)	1.356	0.408	3.321	<0.001	0.401	0.519	0.774	0.439
	Age	-0.222	0.376	-0.803	0.422	0.055	0.388	0.142	0.887
	Year	-1.049	0.426	-2.462	0.014	1.363	0.483	2.821	0.005
	Sample mass	0.353	0.220	1.605	0.108	0.095	0.327	0.291	0.771
	Body mass	-0.262	0.307	-0.855	0.392	-0.455	0.344	-1.322	0.186
	Sex*Age	-0.443	0.355	-1.250	0.211	-0.380	0.774	-0.490	0.624
Trichuris	Intercept	-7.289	2.864	-2.545	0.011 [c]	-	37.347	-2.298	0.022 [d]
prevalence	Sex (ref. female)	1.745	1.632	1.069	0.285	-	-	-	-
	Age	0.043	1.110	0.038	0.969	-2.833	14.324	-0.198	0.843
	Year	0.074	1.414	0.053	0.958	-	-	-	-
	Sample mass	0.275	0.630	0.437	0.662	-	10.797	-2.217	0.027
	Body mass	2.116	1.237	1.711	0.087	-3.930	17.233	-0.228	0.820
	Sex*Age	-0.481	1.403	-0.343	0.732	-	-	-	-
Hymenolepis	Intercept	-1.367	0.710	-1.926	0.054 [e]	0.512	0.797	0.642	0.521 [f]
prevalence	Sex (ref. female)	0.513	0.395	1.299	0.194	-1.395	0.651	-2.145	0.032
	Age	-0.682	0.244	-2.792	0.005	0.029	0.391	0.074	0.941
	Year	0.538	0.446	1.206	0.228	-0.638	0.471	-1.355	0.175
	Sample mass	-0.011	0.205	-0.054	0.957	0.253	0.341	0.744	0.457
	Body mass	1.051	0.339	3.100	0.002	-2.802	1.228	-2.282	0.023
	Sex*Age	-0.374	0.357	-1.048	0.295	2.484	0.991	2.507	0.012

Morphotype richness	Intercept	-0.093	0.329	-0.283	0.777 [g]	0.013	0.393	0.033	0.973 [h]
	Sex (ref. female)	0.603	0.199	3.026	0.002	-0.408	0.269	-1.516	0.130
	Age	-0.245	0.130	-0.892	0.059	-0.157	0.200	-0.787	0.432
	Year	-0.241	0.200	-1.202	0.229	0.428	0.242	1.769	0.077
	Sample mass	0.156	0.101	1.553	0.120	0.132	0.174	0.756	0.450
	Body mass	0.258	0.144	1.796	0.072	-0.431	0.173	-2.496	0.013
	Sex*Age	-0.177	0.170	-1.041	0.298	0.705	0.419	1.682	0.092

[a] N= 235 / 110 (samples/ individuals); $R^2_{marginal}$=0.240, $R^2_{conditional}$=0.240, σ^2<0.001
[b] N= 154/88, $R^2_{marginal}$= 0.201, $R^2_{conditional}$=0.201, σ^2<0.001
[c] N=235 / 110, $R^2_{marginal}$=0.028, $R^2_{conditional}$=0.968, σ^2=95.87
[d] N=154/88, $R^2_{marginal}$=0.014, $R^2_{conditional}$>0.999, σ^2=18241; infections observed only in females in year 2012, hence non-convergence if year and sex appear as fixed terms
[e] N=235 / 110, $R^2_{marginal}$=0.206, $R^2_{conditional}$=0.259, σ^2=0.236
[f] N= 154/ 88, $R^2_{marginal}$=0.147, $R^2_{conditional}$= 0.147, σ^2<0.001
[g] N=235 / 110, $R^2_{marginal}$=0.198, $R^2_{conditional}$=0.260, σ^2=0.076
[h] N=154 / 88, $R^2_{marginal}$ = 0.192, $R^2_{conditional}$= 0.192, σ^2<0.001

Within-individual change in parasite burden with age

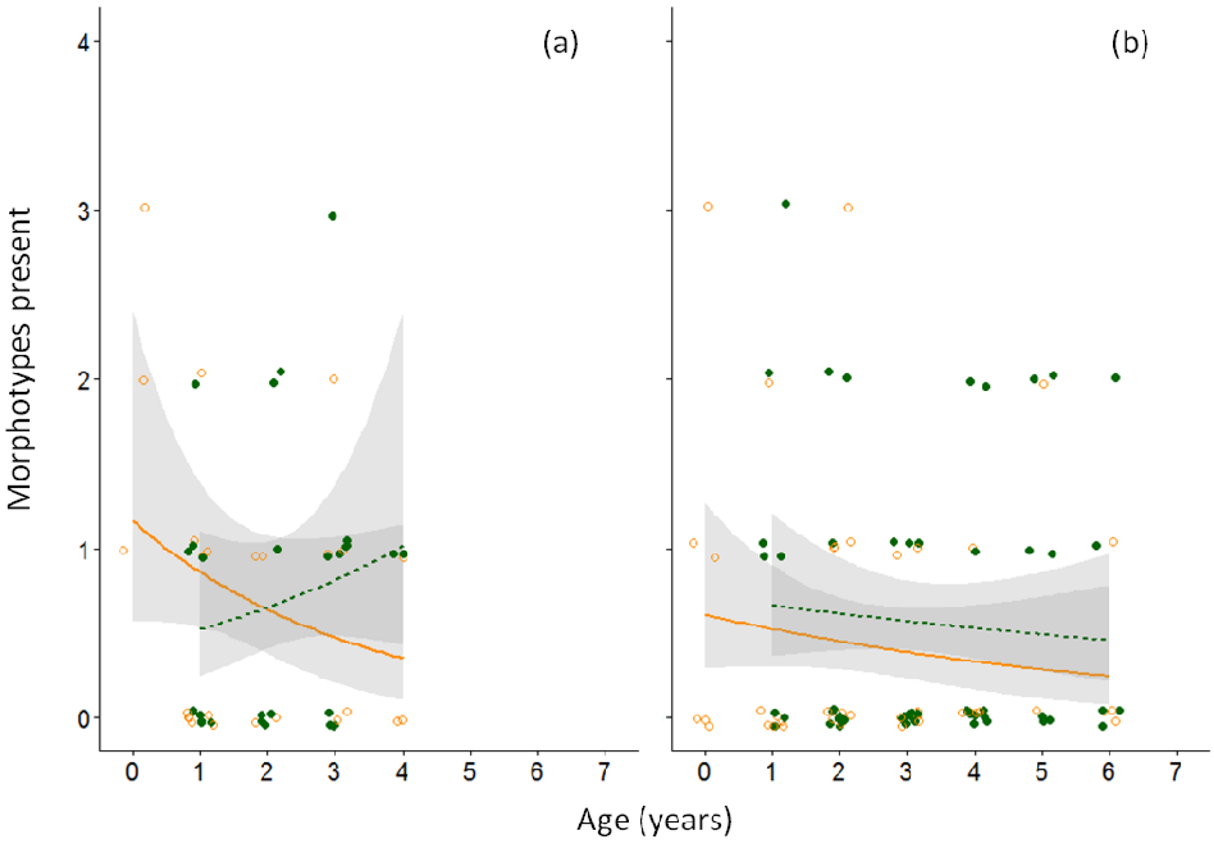

Figure S1: Parasite morphotype richness as a function of age in (a) males and (b) females in the dry season (open orange symbols & solid line) and the rainy season (filled green symbols & dashed line), based on longitudinal data only. A model incorporating the terms age, sex, sample mass and year of sampling suggests an overall declining trend in morphotype richness with age, as found in the larger data set including cross-sectional data. The effect of age on parasite burden is non-significant (Table S3) in these longitudinal data, likely due to the reduced sample size (total N=154 samples/60 individuals).

Table S3: Predictors of parasite burden in males and females based on the longitudinal data, i.e. repeated measures from the same individuals. Predictions are based on the initial models prior to reduction of non-significant terms.

		Males				Females			
		β	SE	z	P	β	SE	z	P
Subulura prevalence	Intercept	-0.525	0.841	-0.624	0.532 [a]	-2.557	1.013	-2.524	0.012 [b]
	Season (ref. dry)	-0.409	1.010	-0.405	0.685	1.628	1.121	1.453	0.146
	Age	-0.639	0.477	-1.340	0.180	-0.482	0.505	-0.956	0.339
	Body mass	0.882	1.062	0.830	0.406	-0.373	1.044	-0.358	0.721
	Season*Age	0.765	0.946	0.809	0.419	0.890	0.685	1.300	0.194
	Season*Body mass	-1.894	1.431	-1.324	0.186	-0.746	1.173	-0.636	0.525
Trichuris prevalence	Intercept	-	-	-	- [c]	-1.940	0.885	-2.192	0.028 [d]
	Season (ref. dry)	-	-	-	-	-0.861	1.234	-0.698	0.486
	Age	-	-	-	-	-0.500	0.431	-1.159	0.247
	Body mass	-	-	-	-	0.281	0.975	0.288	0.773
	Season *Age	-	-	-	-	-	-	-	-
	Season *Body mass	-	-	-	-	-0.742	1.250	-0.593	0.553
Hymenolepis prevalence	Intercept	-1.945	1.311	-1.484	0.138 [e]	-3.233	1.271	-2.544	0.011 [f]
	Season (ref. dry)	0.437	1.373	0.318	0.750	2.312	1.258	1.837	0.066
	Age	-0.949	0.621	-1.528	0.126	0.547	0.542	1.008	0.313
	Body mass	0.325	1.374	0.237	0.813	-1.524	1.145	-1.330	0.183
	Season *Age	1.666	1.105	1.508	0.132	-1.234	0.706	-1.748	0.080
	Season *Body mass	-0.855	1.707	-0.501	0.617	1.254	1.258	0.997	0.319
Morphotype richness	Intercept	-0.464	0.484	-0.958	0.338 [g]	-1.329	0.530	-2.510	0.012 [h]
	Season (ref. dry)	0.087	0.565	0.153	0.878	0.975	0.552	1.765	0.076
	Age	-0.433	0.223	-1.940	0.052	-0.084	0.250	-0.338	0.736
	Body mass	0.176	0.544	0.323	0.747	-0.494	0.528	-0.936	0.349
	Season *Age	0.764	0.492	1.553	0.120	-0.011	0.336	-0.034	0.973
	Season *Body mass	-0.709	0.738	-0.960	0.337	0.096	0.597	0.160	0.873

[a] N= 49/20 samples/ individuals, $R^2_{marginal}$ =0.140, $R^2_{conditional}$= 0.140, σ^2<0.001

[b] N= 95/40 samples/ individuals, $R^2_{marginal}$ =0.189, $R^2_{conditional}$= 0.189, σ^2<0.001

[c] cannot be modeled, see text

[d] N= 95/40 samples/ individuals, $R^2_{marginal}$ =0.184, $R^2_{conditional}$= 0.184, σ^2<0.001; season*age interaction cannot be modeled, see text

[e] N= 49/20 samples/ individuals, $R^2_{marginal}$ =0.134, $R^2_{conditional}$= 0.215 σ^2=0.301

[f] N= 95/40 samples/ individuals, $R^2_{marginal}$ =0.126, $R^2_{conditional}$= 0.243, σ^2= 0.506

[g] N= 49/20 samples/ individuals, $R^2_{marginal}$ =0.161, $R^2_{conditional}$= 0.161, σ^2<0.001

[h] N= 95/40 samples/ individuals, $R^2_{marginal}$ =0.096, $R^2_{conditional}$= 0.142, σ^2=0.064

Predictors of overall parasite infection rate

Overall parasite infection rate (i.e. overall prevalence measured as presence of any intestinal parasite) was examined with season-specific prevalence models using binomial GLMMs. In line with our prediction of earlier immunosenescence in males, a significant sex*age interaction was found in the rainy season with older males exhibiting higher overall infection rates (Table S4). In the dry season, however, parasite infection rate tended to decline more rapidly in males toward old age, leading to a reduced sex difference in infection rate at older age, but this interaction was non-significant (Table S5, Figure S2). Contrary to our prediction of immunosenescence, however, the estimates for the overall effects of age on parasite infection rate was negative in the dry season. In the rainy season, the main effect of age was non-significant when the sex*age interaction was included in the model (Table S4, Figure S2).

Table S4: Predictors of overall infection rate (pooled infection rate by any parasite morphotype) in the dry and rainy season. Presented predictions are based on the reduced model after dropping the terms age*sex and body mass where they had non-significant (P>0.1) effects.

		**β**	**SE**	**z**	**P**
Dry season [a]	Intercept	0.589	0.617	0.955	0.340
	Sex	1.152	0.342	3.367	<0.001
	Age	-0.422	0.182	-2.316	0.021
	Year	-0.110	0.395	-0.278	0.781
	Sample mass	0.419	0.199	2.107	0.035
Rainy season [b]	Intercept	0.916	0.700	1.307	0.191
	Sex	-0.137	0.465	-0.295	0.768
	Age	-0.151	0.344	-0.438	0.662
	Year	0.634	0.407	1.560	0.119
	Body mass	-0.573	0.288	-1.986	0.047
	Sample mass	0.428	0.293	1.458	0.145
	Age*sex	1.563	0.746	2.096	0.036

[a] N=253 samples /113 individuals, $R^2_{marginal}$= 0.151, $R^2_{conditional}$= 0.327, σ^2=0.734

[b] N=154 samples/88 individuals, $R^2_{marginal}$ = 0.132, $R^2_{conditional}$ =0.132, σ^2<0.001

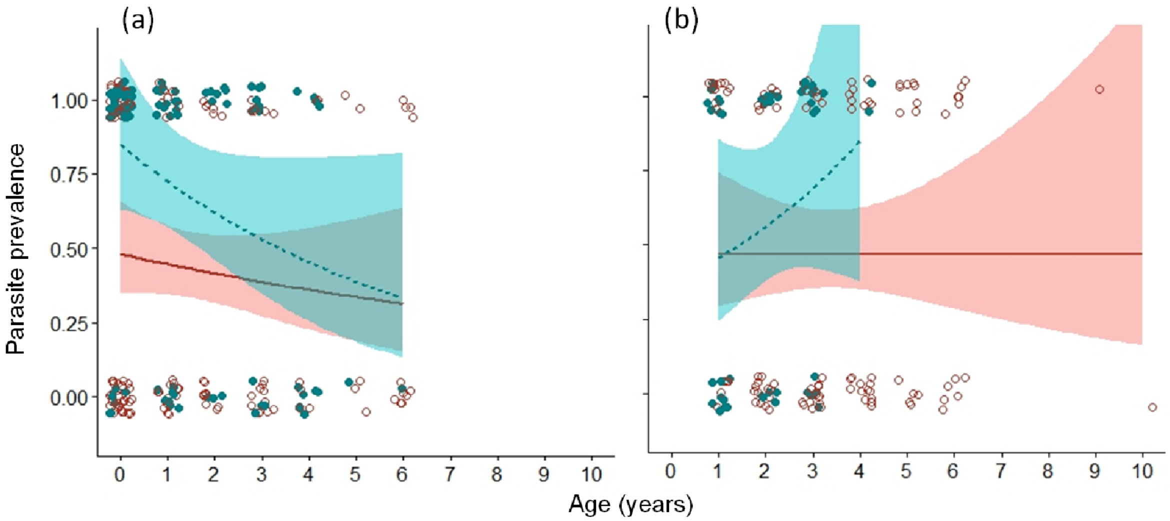

Figure S2: Overall parasite prevalence (pooled prevalence of all morphotypes) in the a) dry and b) rainy season. Shown are all data points (with jitter introduced to the discrete variables for ease of interpretation) for males (solid symbol) and females (open symbol), and loess-smoothed prediction lines and 95% confidence bands for age effects for males (dashed line) and females (solid line).

Table S5: Full models of overall parasite infection rates in the dry and rainy season.

		β	SE	z	P
Dry season [a]	Intercept	0.993	0.707	1.404	0.160
	Sex (Ref. Female)	0.984	0.414	2.378	0.017
	Age	-0.272	0.245	-1.107	0.268
	Year (Ref. 2010)	-0.324	0.429	-0.755	0.450
	Body mass	0.370	0.345	1.071	0.284
	Sample mass	0.441	0.213	2.067	0.039
	Age*sex	-0.681	0.388	-1.754	0.079
Rainy season [b]	Intercept	0.916	0.700	1.307	0.191
	Sex (Ref. Female)	-0.137	0.465	-0.295	0.768
	Age	-0.151	0.344	-0.438	0.662
	Year (Ref. 2011)	0.634	0.407	1.560	0.119
	Body mass	-0.573	0.288	-1.986	0.047
	Sample mass	0.428	0.293	1.458	0.145
	Age*sex	1.563	0.746	2.096	0.036

[a] N= 235 samples/ 110 individuals, $R^2_{marginal}$=0.187, $R^2_{conditional}$=0.352, σ^2=0.706

[b] N= 154 samples/88 individuals, $R^2_{marginal}$=0.132, $R^2_{conditional}$=0.132, σ^2<0.001

Table S6: Predictors of overall parasite prevalence in male and female gray mouse lemurs based on longitudinal data, i.e. repeated measures from the same individuals.

	**Females [a]**				**Males [b]**			
	**β**	**SE**	**z**	**P**	**β**	**SE**	**z**	**P**
Intercept	-1.638	0.465	-3.522	0.001	0.101	0.539	0.187	0.852
Season (ref. dry)	1.733	0.714	2.426	0.015	-0.313	0.740	-0.423	0.672
Age	-0.040	0.237	-0.170	0.865	-0.214	0.357	-0.599	0.549
Body mass	-0.846	0.356	-2.372	0.018	-0.048	0.499	-0.096	0.923

[a] N= 95 samples/40 individuals, $R^2_{marginal}$ =0.110, $R^2_{conditional}$=0.110, σ^2<0.001

[b] N=49 samples/20 individuals, $R^2_{marginal}$ =0.024, $R^2_{conditional}$=0.024, σ^2<0.001

Table S7: Overall parasite prevalence as a predictor of survival to next season. Season reference level is dry season 2010.

		β	SE	z	P
Overall infection [a]	Intercept	-0.013	0.378	-0.035	0.972
	Parasite prevalence (ref. uninfected)	-0.297	0.360	-0.824	0.410
	Sex (ref. female)	-0.017	0.374	-0.045	0.964
	Age	0.012	0.183	0.068	0.946
	season 2012 dry	0.418	0.410	1.021	0.307
	season 2012 rainy	0.978	0.537	1.822	0.068

[a] N=160 individuals, Nagelkerke's R^2= 0.041

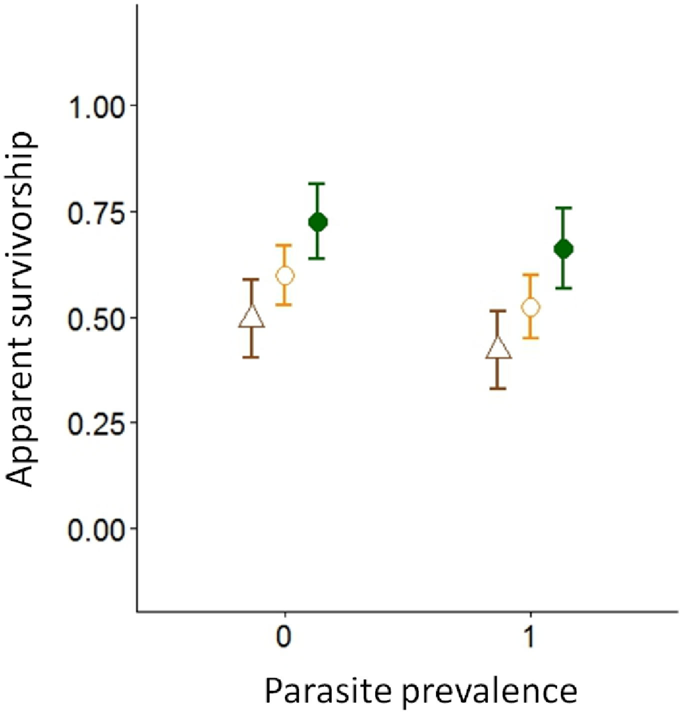

Figure S3: Apparent survival of gray mouse lemurs to next season (~6 months, based on capture data) is not significantly influenced by overall parasite prevalence (Table S7). Shown are season-specific means ±SE for parasite prevalence (infected = 1) in the 2010 dry season (open brown triangle), 2012 dry season (open orange circles) and the 2012 rainy season (filled green circles).

Characteristics of the parasite community of gray mouse lemurs in Kirindy forest

The three common morphotypes of gray mouse lemurs found in the current study concur with a smaller scale study on a different subpopulation in Kirindy forest [1]. The common morphotypes presumably represent both direct (*Trichuris*) and indirect (*Subulura*, *Hymenolepis*) routes of transmission [2]. Of the rare morphotypes found in our study, only *Ascaris* sp. was also identified in an earlier study in Kirindy [1]. Most of the rare egg morphotypes of our study have been described previously for *M. murinus* [2, 3], but identifications have generally not been confirmed via study of adult worms. Hence, the actual taxonomic diversity is unknown and identifications can only be made based on egg morphology, which may at times be inaccurate [2].

The parasite egg morphotypes with prevalence < 10 infected samples each (*Ascaris, Capillaria, Lemuricola,* other *Oxyuridae, Oesophagostomum, Strongylida, Fasciolidae, Metagonimus* and *Opisthorchis* species) were grouped together into a "rare morphotype" response variable and their prevalence analyzed using a binomial GLMM. No statistically significant seasonal differences were found in the prevalence of the rare parasite morphotypes (dry: 7.9%, rainy: 6.0%, $\beta = -0.073$, SE= 0.531, z= -0.138, P= 0.890). None of the predictors in the final model showed association with rare parasite prevalence, nor could the fixed effects in the models explain almost any of the variation in prevalence ($R^2_{marginal}$=0.003-0.006) (Table S8).

Consequently, while most of the recorded rare morphotypes likely represent genuine infections, some of them might be misclassifications due to difficulty of discriminating between closely related taxa based on egg morphology [2]. Although we took every precaution against contamination by heterospecific hosts by cleaning traps in between all captures, occasional contamination might have occurred by feces from other species (e.g. by insects or small reptiles visiting the traps), or undigested eggs of parasites of prey species being deposited in host feces [2].

Table S8: Prevalence of all rare parasite morphotypes in the dry and rainy season.

		**β**	**SE**	**z**	**P**
Dry season [a]	Intercept	-7.034	2.326	-3.024	0.002
	Sex	-0.277	1.434	-0.193	0.847
	Age	-0.273	0.627	-0.436	0.663
	Year	-0.687	1.147	-0.599	0.549
	Sample mass	0.087	0.577	0.151	0.880
Rainy season [b]	Intercept	-10.501	3.113	-3.373	<0.001
	Sex	0.337	1.953	0.173	0.863
	Age	0.396	1.44	0.275	0.783
	Year	-0.16	1.15	-0.139	0.889
	Sample mass	-1.109	1.027	-1.08	0.280

[a] N= 253/ 113/20 (samples/ individuals/ infected), $R^2_{marginal}$=0.003, $R^2_{conditional}$=0.947, σ^2=58.920

[b] N= 217/ 93/13, $R^2_{marginal}$=0.006, $R^2_{conditional}$=0.956, σ^2=70.400

Intensity of overall parasite infection based on fecal egg counts

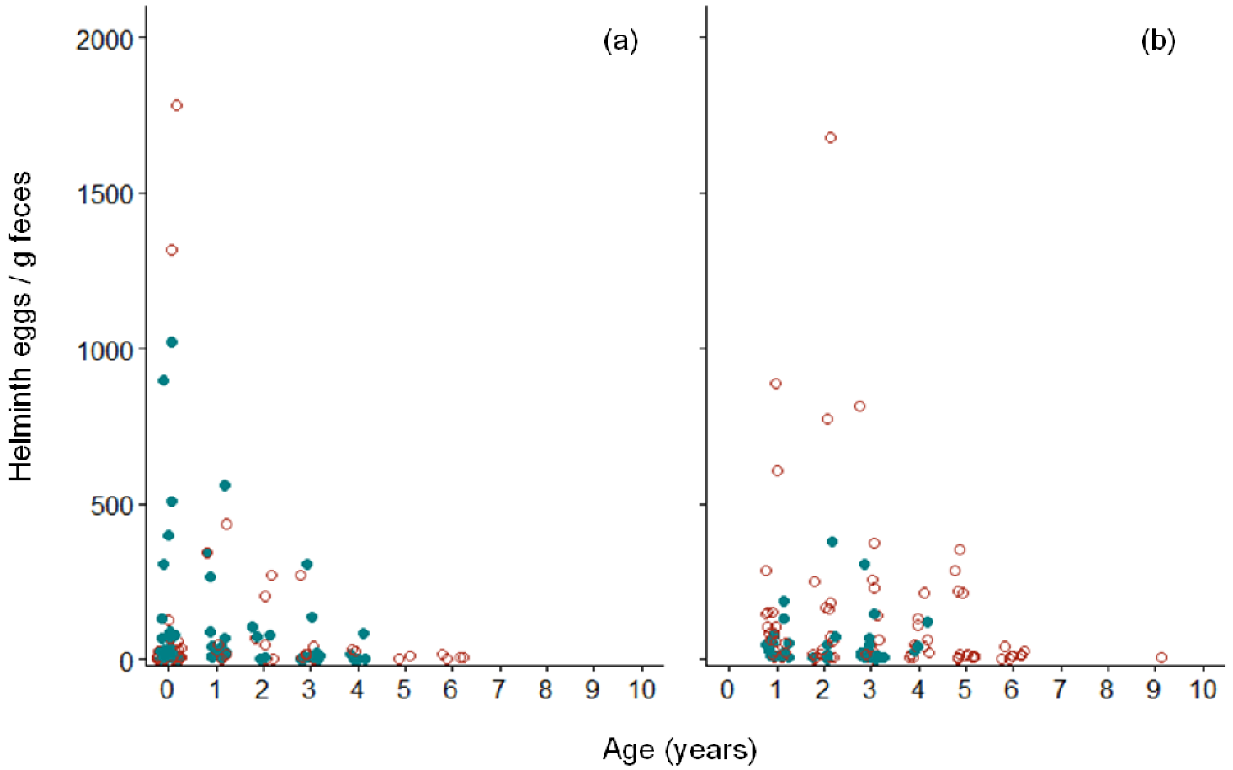

Figure S4: The total number of all helminth eggs recovered in the feces of male (solid blue symbols) and female (open red symbols) gray mouse lemurs as a function of age in the (a) dry and (b) the rainy season. Data are shown for all samples infected with at least one type of parasite.

References

1. Schwensow N, Dausmann K, Eberle M, Fietz J, Sommer S: **Functional associations of similar MHC alleles and shared parasite species in two sympatric lemurs.** *Infection, Genetics and Evolution* 2010, **10:**662-668.

2. Irwin MT, Raharison J-L: **A review of the endoparasites of the lemurs of Madagascar.** *Malagasy Nature* 2009, **2:**66-93.

3. Raharivololona B: **Gastrointestinal parasites of *Cheirogaleus* spp. and *Microcebus murinus* in the littoral forest of Mandena, Madagascar.** *Lemur News* 2006, **11:**31-35.
